# Early switch from intravenous to oral antibiotic therapy in patients with cancer who have low-risk neutropenic sepsis (the EASI-SWITCH trial): study protocol for a randomised controlled trial

**DOI:** 10.1186/s13063-020-04241-1

**Published:** 2020-05-27

**Authors:** Caroline Forde, Ronan McMullan, Mike Clarke, Richard H. Wilson, Ruth Plummer, Margaret Grayson, Cliona McDowell, Ashley Agus, Annmarie Doran, Danny F. McAuley, Anne L. Thomas, Rosemary A. Barnes, Richard Adams, Ian Chau, Vicky Coyle

**Affiliations:** 1 Centre for Cancer Research and Cell Biology, Queen’s University Belfast, Lisburn Road, Belfast, BT9 7AE UK; 2 Centre for Experimental Medicine, Queen’s University Belfast, Belfast, UK; 3 Northern Ireland Methodology Hub, Queen’s University Belfast, Belfast, UK; 4 Northern Ireland Clinical Trials Unit, Belfast Health and Social Care Trust, Belfast, UK; 5 Translational Research Centre, University of Glasgow, Glasgow, UK; 6 Northern Institute for Cancer Research, Newcastle University, Newcastle, UK; 7 Northern Ireland Cancer Research Consumer Forum, Belfast, UK; 8 The Wellcome Wolfson Institute for Experimental Medicine, Queens University Belfast, Belfast, UK; 9 Leicester Cancer Research Centre, Leicester, UK; 10 Cardiff University School of Medicine, Cardiff, UK; 11 Cardiff University and Velindre NHS Trust, Cardiff, UK; 12 The Royal Marsden NHS Foundation Trust, London, UK

**Keywords:** Neutropenic sepsis, Low risk, Oral antibiotics, Randomised controlled trial, Non-inferiority, Cancer

## Abstract

**Background:**

Neutropenic sepsis remains a common treatment complication for patients receiving systemic anti-cancer treatment. The UK National Institute for Health and Care Excellence have not recommended switching from empirical intravenous antibiotics to oral antibiotics within 48 h for patients assessed as low risk for septic complications because of uncertainty about whether this would achieve comparable outcomes to using intravenous antibiotics for longer. The UK National Institute for Health Research funded the EASI-SWITCH trial to tackle this uncertainty.

**Methods:**

The trial is a pragmatic, randomised, non-inferiority trial that aims to establish the clinical and cost-effectiveness of early switching from intravenous to oral antibiotics in cancer patients with low-risk neutropenic sepsis. Patients ≥ 16 years, receiving systemic anti-cancer treatment (acute leukaemics/stem cell transplants excluded), with a temperature of > 38 °C, neutrophil count ≤ 1.0 × 10^9^/L, MASCC (Multinational Association of Supportive Care in Cancer) score ≥ 21 and receiving IV piperacillin/tazobactam or meropenem for less than 24 h are eligible to participate. Patients are randomised 1:1 either (i) to switch to oral ciprofloxacin and co-amoxiclav within 12–24 h of commencing intravenous antibiotics, completing at least 5 days total antibiotics (intervention), or (ii) to continue intravenous antibiotics for at least 48 h, with ongoing antibiotics being continued at the physician’s discretion (control). Patients are discharged home when their physician deems it appropriate. The primary outcome measure is a composite of treatment failures as assessed at day 14. The criteria for treatment failure include fever persistence or recurrence 72 h after starting intravenous antibiotics, escalation from protocolised antibiotics, hospital readmission related to infection/antibiotics, critical care support or death. Based on a 15% treatment failure rate in the control group and a 15% non-inferiority margin, the recruitment target is 230 patients.

**Discussion:**

If the trial demonstrates non-inferiority of early switching to oral antibiotics, with potential benefits for patient quality of life and resource savings, this finding will have significant implications for the routine clinical management of those with low-risk neutropenic sepsis.

**Trial registration:**

ISRCTN: 84288963. Registered on the 1 July 2015. 10.1186/ISRCTN84288963.

EudraCT: 2015-002830-35.

## Background

Neutropenic sepsis is a long-recognised and common complication of systemic anti-cancer treatment (SACT) [1]. The term broadly refers to a significant inflammatory response to a presumed bacterial infection in a person with or without fever and a low blood neutrophil count [2]. Despite the widespread adoption of prophylactic colony-stimulating factors (CSF) and fluoroquinolone antibiotics for patients at high risk of septic complications, neutropenic sepsis remains potentially life threatening. Significant patient morbidity can also occur through hospitalisation and associated dose delays and reductions to planned SACT [2, 3].

Neutropenic sepsis continues to be viewed as a time-critical medical emergency, with widespread agreement that early recognition and prompt administration of broad-spectrum empirical antibiotics are essential to successful treatment [2, 4–6]. However, much less consensus exists on the optimal patient management thereafter, including when to switch from intravenous (IV) to oral antibiotics and the duration of antibiotic treatment and hospital admission. Widely variable practice has been noted among cancer centres in the United Kingdom [7].

A spectrum of neutropenic sepsis severity exists, encompassing a heterogeneous group of patients with variable prognoses [8]. Some patients can quickly deteriorate and develop septic shock from overwhelming infection, for whom ongoing inpatient IV antibiotics with goal-directed resuscitation is critical. At the other end of the spectrum are patients who do not demonstrate clear clinical or microbiological evidence of proven infection, have uncomplicated hospital admissions and are at low risk of developing septic complications. These patients potentially receive overtreatment, with the associated distress of hospitalisation and additional burden to the healthcare system [9]. Risk stratification tools have therefore been developed in an attempt to identify patients predicted to be at low risk of an adverse outcome. The Multinational Association of Supportive Care in Cancer (MASCC) score is the most widely validated risk score for SACT-induced neutropenic sepsis [2, 5, 6, 10] (Table 1).

**Table 1:** Multinational association of supportive care in cancer risk index (adapted from Klastersky (2000)) [10]

Characteristic	Weight
Burden of febrile neutropenia: no or mild symptoms^a^	5
Burden of febrile neutropenia: moderate symptoms^a^	3
No hypotension (systolic blood pressure > 90 mmHg)	5
No chronic obstructive pulmonary disease	4
Solid tumour or no previous fungal infection	4
No dehydration requiring parenteral fluids	3
Outpatient status	3
Age < 60 years	2

The maximum theoretical score is 26

A score of ≥ 21 suggests a low risk of a serious medical complication, including organ failure, critical care support or death

^a^Points attributable to the variable ‘burden of febrile neutropenia’ are not cumulative

A Cochrane review of oral versus IV antibiotics for neutropenic sepsis, evaluating 22 trials, concluded that significant differences are unlikely to exist in treatment failure or mortality rates between oral antibiotic and IV antibiotic strategies. Most studies did not utilise any formal risk stratification tools but excluded high-risk patients with acute leukaemia, haemodynamic instability, evidence of organ failure or localising signs of infection. The Cochrane review therefore broadly supported the early use of oral antibiotics in low-risk neutropenic sepsis but noted that most trials were small in sample size, often were single centre and had methodological concerns. Therefore, a robust recommendation for upfront or early oral antibiotic therapy could not be made [11].

Guidance issued in 2012 by the UK National Institute for Health and Care Excellence (NICE) recommends that switching to oral antibiotics should be considered in patients deemed at low risk of complications after 48 h of IV therapy. This guidance was based on limited evidence supporting the switch to oral antibiotics in low-risk patients but with no consistent time point identified, leading to NICE being unable to recommend an oral switch before 48 h. The NICE guideline development group (GDG) noted that switching at an earlier time point (for example, at 8–16 h) may be beneficial for patients and hospital resource use but that no meaningful evidence currently exists to support this approach [2]. Current European guidelines suggest that after the initial patient assessment, including the prompt institution of empirical broad-spectrum antibiotics, inpatient oral antibiotic treatment may be suitable for patients identified as low risk. However, they note that many clinicians prefer to continue IV treatment for at least 48 h and then consider a change to oral antibiotics if the fever resolves [5].

The NICE GDG also reviewed the evidence for inpatient versus outpatient management of neutropenic sepsis and concluded that outpatient management can be considered for selected low-risk patients, taking into account their individual clinical and social circumstances. Although the meta-regression undertaken by the GDG suggested that early discharge (before 24 h) may be associated with an increased likelihood of readmission or therapy change, the quality of evidence supporting outpatient management was low to moderate. The available data were limited by a lack of reporting of key outcomes such as critical care admission or clinically documented infection and a very low event rate for adverse outcomes including death [2]. Similarly, the literature relating to the impact on the quality of life for different models of care, including immediate use of oral antibiotics and non-admission to hospital, is negligible, with a single study suggesting that role function improved more for inpatients than for home care patients but that emotional function declined with hospital admission [12]. Therefore, if a short period of hospital admission was found to be safe and effective for selected patients with neutropenic sepsis, this approach could provide considerable improvements in quality of life and health resource usage.

NICE therefore recommended that a randomised trial should be undertaken to evaluate the effectiveness of stopping IV antibiotics or switching to oral antibiotics within the first 24 h of treatment in patients receiving IV antibiotics for neutropenic sepsis. The early switch to oral antibiotic therapy in patients with low-risk neutropenic sepsis (EASI-SWITCH) trial was developed in response to this recommendation and to a commissioned call from the UK National Institute of Health Research (NIHR) Health Technology Assessment (HTA) programme to address this evidence gap. It aims to establish the clinical and cost effectiveness of an early switch to oral antibiotics 12–24 h after IV antibiotic treatment commences in low-risk cancer patients with neutropenic sepsis.

### Study hypothesis and objectives

An early switch to oral antibiotic therapy, 12–24 h after IV antibiotic treatment commences in low-risk cancer patients with neutropenic sepsis, is hypothesized as non-inferior to standard care. The primary objective is to determine whether an early switch to oral antibiotic therapy is non-inferior to current standard care of IV antibiotics for at least 48 h in terms of treatment failure. Treatment failure is defined by a composite measure incorporating a number of important clinical outcomes assessed at day 14.

A number of secondary objectives are included to assess the impact of an early switch to oral antibiotics on patients and the health service. Secondary objectives are (i) short-term change in health-related quality of life, (ii) treatment cost-effectiveness, (iii) time to fever resolution from initial IV antibiotic administration, (iv) adverse events related to the antibiotics, (v) total length of hospital stay, (vi) readmission to hospital within 28 days, (vii) death within 28 days, (viii) adjustment to subsequent scheduled cycle of chemotherapy within 28 days and (ix) patient preference for antibiotic treatment strategy. As an exploratory objective, whole blood, serum and plasma samples are also being collected for the future analysis of potential biomarkers, which may enhance neutropenic sepsis risk stratification and management.

## Methods/Design

### Study design

**Fig. 1:**
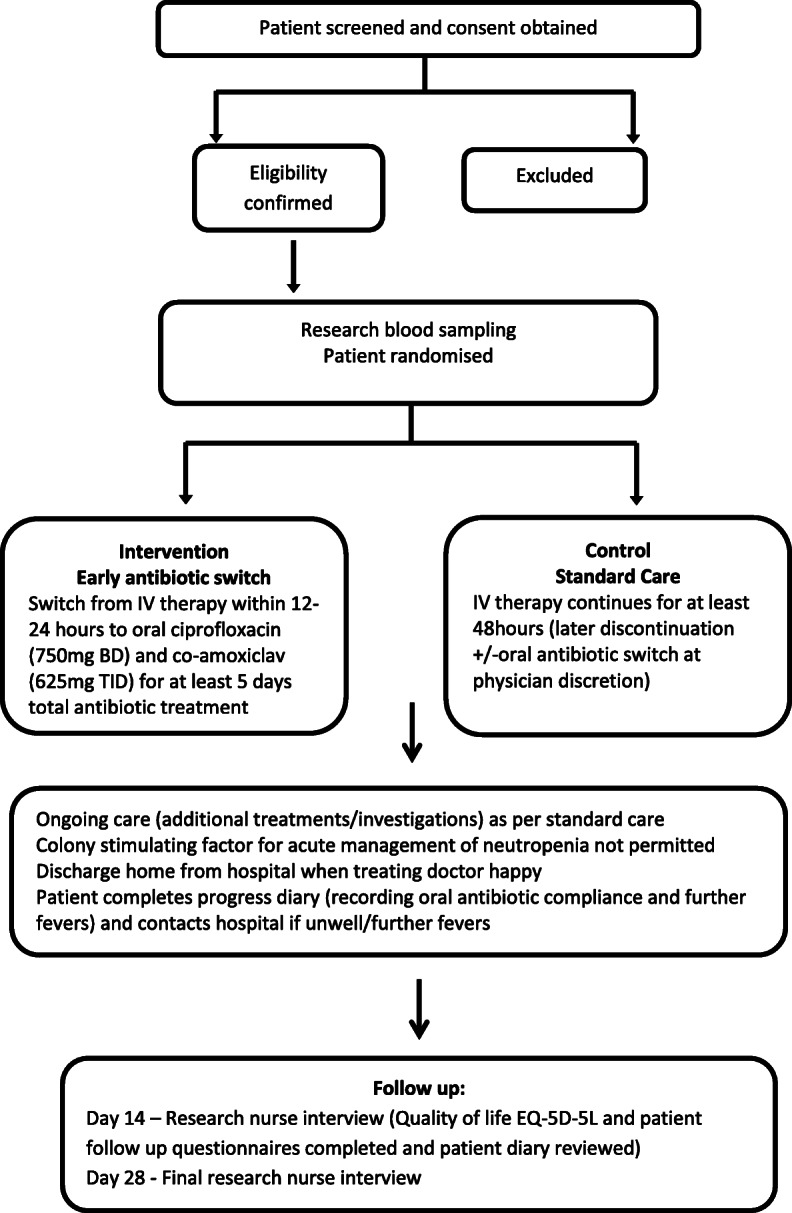
Study schematic diagram

A pragmatic, randomised, open-label, non-inferiority trial has been designed to compare the early switch to oral antibiotics, 12–24 h after IV antibiotic treatment commences, versus standard care, which comprises IV treatment for at least 48 h, in cancer patients with neutropenic sepsis at low risk of complications (Fig. 1). The EASI-SWITCH trial is funded by the UK NIHR HTA programme, sponsored by the Belfast Health and Social Care Trust and supported by the Northern Ireland Clinical Trials Unit (NICTU). The study protocol (most recently Version 8.0, 21 June 2018) was developed and implemented in accordance with the Consolidated Standards of Reporting Trials (CONSORT) 2010 statement [13] and the Standard Protocol Items: Recommendations for Interventional Trials (SPIRIT) 2013 checklist [14], as provided in Additional file 1.

### Study setting

Participants are recruited from NHS cancer centres and units across England, Scotland, Wales and Northern Ireland, to ensure that the sample is broadly representative of patients developing neutropenic sepsis across the United Kingdom. A list of sites can be obtained from the NICTU.

### Eligibility criteria

Eligibility is determined by the inclusion and exclusion criteria listed in Table 2. Patients enrolled in other Phase I Investigational Medicinal Product (IMP) studies and other antimicrobial IMP studies are excluded. Patients enrolled in other Phase II-IV IMP or observational studies are potential candidates for this study if the burden of participating is not expected to be too onerous. Based on NICE guidance, all patients enrolled have already been commenced on standard dose IV piperacillin/tazobactam or meropenem as initial antibiotic treatment, in accordance with the local neutropenic sepsis policy and the summary of product characteristics for these antibiotics.

**Table 2:** Current trial eligibility criteria

Eligibility criteria	
**Inclusion criteria**	
(i)	Age over 16 years
(ii)	Receiving SACT for a cancer diagnosis
(iii)	Started on empirical IV piperacillin/tazobactam or meropenem, for suspected NS, for less than 24 h. Patients who have been started on additional antimicrobial drugs (e.g., gentamicin or teicoplanin) are eligible, provided the physician in charge of their care is willing to stop this additional antimicrobial at the time of enrolment
(iv)	Absolute neutrophil count ≤ 1.0 × 109/L with **either** a Temperature of at least 38 °C **or** Other signs or symptoms consistent with clinically significant sepsis, e.g., hypothermia. Self-measurement at home or earlier hospital assessment of temperature are acceptable provided this is documented in the medical notes and is within 24 h prior to IV antibiotics administration.
(v)	Expected duration of neutropenia < 7 days
(vi)	Low risk of complications using a validated risk score (MASCC ≥ 21)
(vii)	Able to maintain adequate oral intake and take oral medication
(viii)	Adequate hepatic (AST+/ALT < 5x upper limit of normal (ULN)) and renal function (serum creatinine < 3x ULN) within the 24 h prior to randomisation
(ix)	Physician in charge of care willing to follow either the intervention or standard care protocol per randomisation, at enrolment, including **not** treating with colony-stimulating factor (CSF). Prophylactic use of CSF is not an exclusion criteria if prescribed routinely as an integral component of a specific SACT regimen.
**Exclusion criteria**	
(i)	Underlying diagnosis of acute leukaemia or haematopoietic stem cell transplant
(ii)	Hypotension (systolic pressure < 90 mmHg or reduction of > 40 mmHg from known baseline on > 1 measurement) within the 24 h prior to randomisation
(iii)	Prior allergy, serious adverse reaction, or contraindication to any study drug
(iv)	Enrolled in the trial with prior episode of neutropenic sepsis
(v)	Previously documented as being colonised with an organism resistant to a study drug regimen, e.g., MRSA (methicillin-resistant *Staphylococcus aureus*)
(vi)	Localising signs of severe infection (pneumonia, soft tissue infection, central venous access device infection, presence of purulent collection)
(vii)	Patient unable to provide informed consent
(viii)	Pregnant or breastfeeding women

### Standard care (control arm)

Participants in the standard care group are allocated to continue treatment with IV antibiotics for a minimum of 48 h. This was selected based on the NICE guidance recommendations [2]. Subsequent antibiotic management is at the discretion of the treating physician, who may switch to oral antibiotics or stop antibiotics at any point thereafter, thereby reflecting the variation encountered in routine clinical practice [7].

### Early oral antibiotic switch (intervention arm)

Participants randomised to the intervention group switch from IV antibiotic treatment within 12–24 h of starting, to co-amoxiclav 625 mg three times daily and ciprofloxacin 750 mg twice daily, to complete at least 5 days of antibiotic treatment in total. The timepoint of the oral switch is defined as the time at which the final dose of IV antibiotic is administered. The combination of a quinolone and a second drug active against gram-positive bacteria (e.g., co-amoxiclav) was based on the conclusions of the Cochrane review [11].

Use of CSFs as treatment for the episode of neutropenic sepsis is prohibited in both intervention groups. Prophylactic CSF is not an exclusion criterion if prescribed routinely as an integral component of a specific SACT regimen. Any other additional treatments or investigations that patients require are as per standard care. Escalation from protocol-specified antibiotic treatment might be required if evidence emerges of clinical deterioration, progression of the presumed infection, a microbiologic indication based on positive cultures or an adverse reaction to the prescribed antibiotics. A change from protocol-specified antibiotics, including additional antibiotic treatment other than the study drugs or persistent/recurrent fever (> 38 °C) after 72 h is within the definition of treatment failure, with such participants reaching the trial’s primary endpoint.

Patients are discharged home from hospital when their treating physician is content to do so, with a patient diary to record any further temperatures and oral antibiotic compliance. Due to the pragmatic nature of the trial, specific discharge criteria have not been protocolised, but clinicians are assumed to take into consideration the patient’s overall clinical condition and psycho-social circumstances, as per their normal clinical practice.

The oral and IV study antibiotics (oral ciprofloxacin and co-amoxiclav and IV piperacillin/tazobactam and meropenem) are regarded as IMPs by the Medicines and Healthcare products Regulatory Agency (MHRA) for the purposes of this trial. EASI-SWITCH has been designated a Type A study with no higher risk than the risk of standard medical care, and therefore, as all are UK licensed drugs, routine hospital stock is being used, supplied and labelled in accordance with usual clinical practice.

### Primary outcome measure

The primary outcome measure of treatment failure is assessed at day 14. This composite measure is defined by the presence of any one of (i) persistence or recurrence of fever (temperature > 38 °C) after 72 h of IV antibiotic initiation, (ii) physician-directed escalation from protocol-specified antibiotic treatment, (ii) re-admission to hospital (related to infection or antibiotic treatment), (iv) admission to critical care or (iv) death.

A core outcome set is unfortunately lacking for neutropenic sepsis. The constituents of our composite were determined from the NIHR commissioning brief, international expert consensus guidelines [15, 16, 17], other large neutropenic sepsis reported trials [18] and feedback from patient representatives and co-investigators on outcomes that are important to patients.

### Secondary outcome measures

A number of secondary outcome measures address the secondary objectives as described above and have been selected to support the assessment of the clinical and cost-effectiveness of an early oral antibiotic switch. The following are assessed on day 14:

(i) Time to resolution of fever from initial IV antibiotic administration
(ii) Adverse events due to antibiotics or their route of administration
(iii) Hospital resource utilisation, including the length of hospitalisation
(iv) Health-related quality of life (based on the EQ-5D-5 L measurement tool and compared with EQ-5D-5 L from baseline) [19]
(v) Cost-effectiveness. The cost- effectiveness analysis, consistent with the primary outcome measure, will be performed to estimate the cost per treatment failure avoided at 14 days, and a cost-utility analysis will estimate the cost per quality-adjusted life year (QALY) at 14 days.
(vi) Patient preferences for antibiotic treatment (using a trial specific discrete choice questionnaire based on a previously published neutropenic sepsis study in haematological malignancy) [20].

Further secondary outcomes, comprising readmission to hospital (related to infection or antibiotic treatment), change in subsequent planned SACT and death, are also assessed on day 28.

### Participant timeline

Two follow-up reviews occur with a research nurse, by telephone if convenient, at day 14 and day 28. At day 14, the primary outcome measure data are collected with patients assessed for evidence of treatment failure. Patients complete two short questionnaires for health-related quality of life (EQ-5D-5 L) and antibiotic treatment preference (trial-specific patient follow-up questionnaire) and review their completed diaries. A shorter review at day 28 captures survival status, adverse events, hospital admissions and the impact of the neutropenic sepsis episode on their next SACT cycle (if applicable). A summary schedule of assessments is detailed in Table 3.

**Table 3:** Schedule of assessments

	Study period						
			Day 0	Study visits and procedures			
	Pre-consent (standard care)	Pre-randomisation	Randomisation	Day 1–2	Day 3–5	Day 6–14	Day 28
**Pre-consent eligibility screening**							
Eligibility screening as appropriate (as per standard care), e.g., ANC, AST/ALT, creatinine, blood culture. When available, Hb, platelets, CRP, albumin, lactate	**X**						
**Informed consent**							
Informed consent obtained		**x**					
**Pre-randomisation eligibility and assessments**							
Eligibility screening as appropriate (non-standard care) e.g., pregnancy test, MASCC score, max temp prior to randomisation, signs/symptoms of sepsis		**x**					
EQ-5D-5 L		**x**					
**Randomisation**							
Standard care antibiotic administration	**X**	**x**	**x**	**x**	**x**		
Intervention (early switch) antibiotic administration *(IV antibiotics will commence prior to informed consent)*	**X**	**x**	**x**	**x**	**x**		
Research blood sample			**x**				
GP letter sent			**x**				
**Baseline assessments to be completed on CRF after eligibility is confirmed**							
Demographics, vital signs, cancer, medical and SACT history, hospital admission details		**x**					
Concomitant medications		**x**	**x**	**x**	**x**	**x**	
Relevant microbiological results		**x**	**x**	**x**	**x**	**x**	
**Daily data collection**							
Antibiotic regimen	**X**	**x**	**x**	**x**	**x**	**x**	
Highest daily temperature *(whilst inpatient or temperature recorded if unwell as an outpatient)*	**X**	**x**	**x**	**x**	**x**	**x**	
**Protocol compliance**							
Adherence to protocol specified intervention				**x**	**x**		
**Patient follow-up**							
Survival status						**x**	**x**
EQ-5D-5 L						**x**	
Patient follow-up questionnaire						**x**	
New medications						**x**	**x**
Changes to next planned SACT cycle							**x**
Hospital discharge/re-admission/critical care admission details						**x**	**x**
Recording and reporting of adverse events						**x**	

### Sample size

The target sample size is 230 patients. This is based on an assumed 15% treatment failure rate in the standard care arm and 15% non-inferiority margin, at 90% power (one-sided 97.5% confidence intervals (CI)), which requires 98 patients per group. A 5% dropout rate and 10% crossover from the control to intervention group have also been accounted for, resulting in the target of 115 participants per group (230 in total).

The assumed 15% treatment failure rate for patients receiving standard care was derived from the three studies thought to best reflect the proposed control arm in relation to the populations included and the duration of the IV antibiotic treatment administered [20–22].

Selecting the non-inferiority margin was challenging due to the limited evidence available to help guide this selection. A 10% non-inferiority margin was originally chosen to reflect the recommendations from a historic consensus guideline for neutropenic antibiotic trials [15]. This guidance relates to the overall neutropenic sepsis population but with no consideration for stratification by risk of septic complications and therefore may be overly simplistic given the significant differences in clinical outcomes of ineffective treatment in low- and high-risk patients. The trial’s target population are patients at low risk of septic complications and are selected through their MASCC score and additional study eligibility criteria. Treatment failure in this low-risk population, as highlighted in the Cochrane review, therefore typically results in persistence or recurrence of fever, leading to prolongation of admission or readmission for further IV antibiotic treatment, with no association noted between mortality and oral antibiotic treatment in low-risk patients [11]. Given that the study population were patients with neutropenic sepsis at low risk of complications, UK oncologists and patient representatives were surveyed and supported increasing the non-inferiority margin from 10 to 15%. Respondents noted that, even if the treatment fails for up to an extra 15 per 100 patients as a result of the intervention (in addition to the expected 15 treatment failures), this would be greatly outweighed by the advantage of 70 patients having successful oral treatment, often at home.

### Recruitment

Patients commenced on IV antibiotics for neutropenic sepsis at each study site are screened daily for eligibility within 24 h of starting the IV treatment. Informed consent is obtained from each participant by trained research staff (Additional files 2 and 3). Consent also is sought for the blood samples to be taken, stored and analysed for this study and for future ethically approved research. Consideration has been given to the short window for patients to consider trial participation and provide informed consent and strategies taken to raise trial awareness at the sites, which include displaying patient information posters in clinical areas and making a short patient information sheet available to patients at high risk of neutropenic sepsis.

### Randomisation and allocation concealment

Eligible patients are randomised using an automated system, with randomly permuted blocks, 1:1 to the intervention and standard care groups. This use of an automated randomisation system and restricting access to the randomisation sequence to only the trial statistician ensures that allocation concealment is maintained.

### Blinding

Due to the pragmatic nature of this trial, participants, care providers and the research team are not blinded to the allocated treatment. Furthermore, patient representatives advised that participants were highly likely to reveal their treatment allocation to outcome assessors, and therefore, any attempt to blind this group would also be subverted.

### Data collection methods and management

Trial data, including study specific worksheets, patient diaries and the two patient questionnaires (EQ-5D-5 L and patient antibiotic treatment preference questionnaire) are entered onto a web-based case report form on a clinical trial database (MACRO) by delegated site personnel. Data are processed as per the study specific data management plan. Patient confidentiality is maintained with patients only identified by their assigned unique trial identifier and initials, and all databases are password protected. On-site monitoring visits are conducted by NICTU in accordance with the trial monitoring plan at each site, from the time of site initiation until close-out. Compliance with the protocol and the implemented amendments is carefully reviewed. All protocol amendments to date have received the appropriate regulatory and ethical approvals prior to electronic dissemination to investigators with appropriate training provided by NICTU.

### Proposed statistical analyses

An intention to treat analysis (regardless of protocol adherence) as well as a per protocol analysis (involving only those patients completing their originally allocated treatment) will be conducted. Non-inferiority of early switch will only be proven if it is demonstrated in both analyses, to minimize the risk of bias wrongly concluding non-inferiority. Analyses will be one-sided and at a significance level of 0.05. The difference in treatment failure rate (95% CI) will be compared to the non-inferiority margin of 15%. As this is a non-inferiority trial, the null hypothesis is that the degree of inferiority of the intervention to the control (standard care) is greater than the non-inferiority margin of 15%. The alternative hypothesis is therefore that the intervention is inferior to the control by less than the non-inferiority margin of 15%. Therefore, non-inferiority would be established by showing that the upper bound of a one-sided 95% CI for control-intervention does not exceed 15%.

Exploratory subgroup analyses will be reported using 99% CI. Logistic regression will be used with interaction terms for the following subgroups: (i) tumour type (solid tumour vs. lymphoma), (ii) neutrophil count at randomisation (≤ 0.5 × 10^9^/L vs. 0.5–1.0 × 10^9^/L) and (iii) maximum temperature at randomisation (< 38 °C vs. ≥ 38 °C).

Baseline characteristics, follow-up measurements and safety data will be described using appropriate descriptive summary measures. A within-trial economic evaluation will be performed to assess the cost-effectiveness of an early switch to oral antibiotics compared with standard care. A cost-effectiveness analysis consistent with the primary outcome measure will be carried out to estimate the cost per treatment failure avoided at day 14 and a cost utility analysis will estimate the cost per QALY at day 14.

### Monitoring

A number of oversight committees have been established for EASI-SWITCH. These include a Trial Management Group, responsible for the day-to-day operational management and a Trial Steering Committee, of which 75% of the members are independent from the trial team, providing overall supervision of the trial’s progress. An independent Data Monitoring and Ethics Committee (DMEC) comprises experts in the field and is monitoring the overall conduct of the trial as well as relevant data, ensuring that the rights, safety and wellbeing of trial participants are being safeguarded. The DMEC charter details the terms of reference of the DMEC, including membership and responsibilities, and is available from the NICTU. Only adverse reactions (adverse events related to the administration of study drugs) and serious adverse events are currently being recorded and reported. All deaths occurring within 28 days of randomisation will be reported as a serious adverse event regardless of the underlying pathology. No specific post-trial provisions have been made for the participants. The sponsor provides indemnity for any negligent harm caused to patients by the design of the trial protocol through the Clinical Negligence Fund in Northern Ireland. The study will be stopped early if recommended by the Trial Steering committee, sponsor, regulatory authorities or research ethics committee.

### Dissemination

The study findings will be published in international peer-reviewed journals and presented at both national and international meetings and to appropriate patient groups, with authorship granted to those who have made a substantive intellectual contribution to the study. Requests for data sharing will be reviewed on an individual basis by the chief investigators and trial management group.

## Discussion

SACT use continues to rise steadily, and therefore, neutropenic sepsis will likely continue to represent a common and potentially life-threatening treatment complication for cancer patients. NICE guidance in 2012 provided the first UK consensus guideline, providing robust evidence-based recommendations aimed at unifying and improving neutropenic sepsis practice, but importantly, this guidance also highlighted a number of outstanding questions regarding optimal management. NICE were unable to recommend switching from empirical IV to oral antibiotics at less than 48 h in low-risk patients because of the uncertainty about whether this achieved comparable outcomes to current standard practice of longer IV antibiotics [2]. To bridge this evidence gap, the EASI-SWITCH trial commissioned by the UK NIHR aims to provide a definitive randomised trial evaluating an early oral switch in low-risk neutropenic sepsis patients.

The EASI-SWITCH trial was designed with the intention of delivering a rigorous but highly pragmatic trial, which will accurately assess the effectiveness of an early oral switch in routine clinical practice. The generalisability of the trial’s results and their potential to inform clinician practice and neutropenic sepsis policies has been a priority during protocol development. The trial team acknowledges that, even with the introduction of NICE guidance, variation remains in local neutropenic sepsis policies and clinicians’ individual practices throughout the UK with respect to criteria for diagnosing neutropenic sepsis, routine use of risk stratification tools and approaches to antibiotic management and hospital admission. Whilst most centres and clinicians have retained a cautious approach, which reflects the current NICE guidance, some have embraced novel strategies to stratify risk and manage low-risk patients on oral antibiotics as outpatients.

The trial’s eligibility criteria and protocolised treatment in the standard care (control) group aim to best reflect the management of the majority of low-risk patients being commenced on a neutropenic sepsis treatment pathway in routine practice and the treatment they receive. Apart from protocolising the minimum requirements for IV antibiotics in the control group and the oral antibiotic intervention, we have strived to give physicians flexibility with all other aspects of patients management, including ongoing antibiotic and discharge decisions, as would be occurring in routine practice. The outcomes have also been carefully selected to focus on those issues most relevant to patients and clinicians for experiencing and delivering high quality, safe and effective neutropenic sepsis care. This has resulted in as pragmatic a trial design as possible, which aims to address some of the previous limitations of trials in this field and provide results that will be generalisable to the majority of UK practice and therefore help inform routine clinical care and policies across the NHS.

An internal pilot study preceded the main trial, with the main parameters of interest being recruitment rates, adherence to the protocol-specified intervention and separation in terms of timing of the antibiotic switch between the two intervention groups. The pilot phase involved four sites and lasted 9 months. Adherence to the protocol-specified intervention in both intervention groups was acceptable (> 75%), and adequate separation existed between the groups, in terms of the timing of the antibiotic switch being at least 24 h between them. On the other hand, recruitment rates were lower than anticipated. Adjustments were therefore made to the eligibility criteria to align even more fully with NICE guidance and clinical practice. This included broadening the diagnostic criteria for neutropenic sepsis, definitions for liver dysfunction and hypotension and permitting recent prophylactic antibiotic use. The adjusted eligibility criteria positively impacted recruitment, and we proceeded to the main trial, expanding the number of participating sites. However, an overall downward trend in neutropenic sepsis admissions at sites resulted in the target recruitment rate per site being lowered and the number of recruiting sites being further increased. An adjustment to the non-inferiority margin and therefore sample size was also undertaken as described above to ensure delivery of a clinically meaningful study.

If EASI-SWITCH meets its primary outcome, demonstrating that an early switch to oral antibiotics in low-risk patients with neutropenic sepsis is non-inferior to current treatment, it will provide evidence for an early oral switch as a management strategy for such patients. Treatment of low-risk neutropenic sepsis based on oral therapy could bring advantages for both patients and the NHS. Such an approach could be expected to provide quality of life benefits for patients, with improved convenience from a shorter length of hospital stay and less IV access complications, including infection. Potential resource benefits also exist for healthcare services by reducing healthcare utilisation, including drug costs, aseptic preparation and administration time, as well as inpatient treatment costs.

## Trial status

Recruitment to the initial pilot study commenced February 2016. At the time of the initial journal submission, (October 2019), recruitment was ongoing across 15 UK sites using protocol version 8 (21 June 2018). At the end of November 2019, a decision was taken to halt recruitment to the trial based on a DMEC recommendation to stop on the grounds of slow recruitment. No safety concerns existed to continue protocol-directed therapy and follow-up of all enrolled patients, with a current plan for database lock in May 2020.

The EASI-SWITCH team can be contacted at the Northern Ireland Clinical Trials Unit at EASI-SWITCH@nictu.hscni.net.

## Supplementary information

**Additional file 1.** SPIRIT checklist.

**Additional file 2.** Patient information sheet.

**Additional file 3.** Patient consent form.

## Data Availability

Requests for data generated or analysed during this current study will be reviewed on an individual basis by the chief investigators (Dr. Coyle and Dr. McMullan) and the trial management group.

## Abbreviations

CI: confidence interval
CONSORT: Consolidated Standards of Reporting Trials
CSF: colony-stimulating factor
GDG: guideline development group
HTA: health technology assessment
IMP: investigational medicinal product
IV: intravenous
MASCC: Multinational Association of Supportive Care in Cancer
MHRA: Medicines and Healthcare Products Regulatory Agency
NICE: National Institute for Health and Care Excellence
NICTU: Northern Ireland Clinical Trials Unit
NIHR: National Institute for Health Research
QALY: quality-adjusted life year
SACT: systemic anti-cancer treatment
SPIRIT: Standard Protocol Items: Recommendations for Interventional Trials

## References

[1] Schimpff S, Satterlee W, Young VM, Serpick A (1971). Empiric therapy with carbenicillin and gentamicin for febrile patients with cancer and granulocytopenia. N Engl J Med.

[2] The National Institute for Health and Care Excellence (2012). Clinical guideline. Neutropenic sepsis: prevention and management of neutropenic sepsis in cancer patients.

[3] Herbst C, Naumann F, Kruse EB, Monsef I, Bohlius J, Schulz H, Engert A. Prophylactic antibiotics or G-CSF for the prevention of infections and improvement of survival in cancer patients undergoing chemotherapy. Cochrane Database Syst Rev. 2009;(1):CD007107. 10.1002/14651858.CD007107.pub2 19160320

[4] National Chemotherapy Advisory Group. Chemotherapy services in England: ensuring quality and safety a report from the National Chemotherapy Advisory Group: NCAG; 2009. https://webarchive.nationalarchives.gov.uk/20130104173757/http://www.dh.gov.uk/en/Publicationsandstatistics/Publications/DH_104500. Accessed 5 Jan 2020.

[5] Klastersky J, de Naurois J, Rolston K, Rapoport B, Maschmeyer G, Aapro M, Herrstedt J (2016). Management of febrile neutropaenia: ESMO clinical practice guidelines. Ann Oncol.

[6] Taplitz R, Kennedy EB, Bow EJ, Crews J, Gleason C, Hawley DK (2018). Outpatient management of fever and neutropenia in adults treated for malignancy: American Society of Clinical Oncology and Infectious Diseases Society of America Clinical Practice Guideline Update. J Clin Oncol.

[7] Simmons T (2012). An assessment of need. Neutropenic sepsis: prevention and management of neutropenic sepsis in cancer patients.

[8] Klastersky J, Paesmans M (2013). The Multinational Association for Supportive Care in Cancer (MASCC) risk index score: 10 years of use for identifying low-risk febrile neutropenic cancer patients. Support Care Cancer.

[9] Rubenstein EB, Rolston K, Benjamin RS, Loewy J, Escalante C, Manzullo E (1993). Outpatient management of febrile episodes in low risk neutropenic patients with cancer. Cancer..

[10] Klastersky J, Paesmans M, Rubenstein EB, Boyer M, Elting L, Feld R (2000). The Multinational Association for Supportive Care in Cancer risk index: a multinational scoring system for identifying low-risk febrile neutropenic cancer patients. J Clin Oncol.

[11] Vidal L, Ben Dor I, Paul M, et al. Oral versus intravenous antibiotic treatment for febrile neutropenia in cancer patients. Cochrane Database Syst Rev. 2013;(10):CD003992. 10.1002/14651858.CD003992.pub3 PMC6457615 24105485

[12] Talcott J, Clark J, Siegel R, Loggers ET, Lu C, Godley P (2011). Safety of early discharge for low-risk patients with febrile neutropenia: a multicenter randomized controlled trial. J Clin Oncol.

[13] Schulz KF, Altman DG, Moher D, for the CONSORT Group (2010). CONSORT 2010 Statement: updated guidelines for reporting parallel group randomised trials. BMJ..

[14] Chan AW, Tetzlaff JM, Altman DG, Laupacis A, Gotzsche PC (2013). Krieza-Jeric et al. SPIRIT 2013 statement: defining standard protocol items for clinical trials. Ann Intern Med.

[15] Feld R, Paesmans M, Freifeld AG, Klastersky J, Pizzo PA, Rolston KV (2002). Methodology for clinical trials involving patients with cancer who have febrile neutropenia: updated guidelines of the Immunocompromised Host Society/Multinational Association for Supportive Care in Cancer, with emphasis on outpatient studies. Clin Infect Dis.

[16] Herdman M, Gudex C, Lloyd A, Janssen MF, Kind P, Parkin D (2011). Development and preliminary testing of the new five-level version of the EQ-5D (EQ-5D-5L). Qual Life Res.

[17] Hughes WT, Pizzo PA, Wade JC, Armstrong D, Webb CD, Young LS (1992). Evaluation of new anti-infective drugs for the treatment of febrile episodes in neutropenic patients. Infectious Diseases Society of America and the Food and Drug Administration. Clin Infect Dis.

[18] Cometta A, Zinner S, Bock R, Calandra T, Gaya H, Kastersky J (1995). Piperacillin-tazobactam plus amikacin versus ceftazidime plus amikacin as empiric therapy for fever in granulocytopenic patients with cancer. The International Antimicrobial Therapy Cooperative Group of the European Organization for Research and Treatment of Cancer. Antimicrob Agents Chemother.

[19] Lathia N, Isogai P, Walker S, De Angelis C (2013). Eliciting patients’ preferences for outpatient treatment of febrile neutropenia: a discrete choice experiment. Support Care Cancer.

[20] Innes HE, Smith DB, O’Reilly SM, Clark PI, Kelly V, Marshall E (2003). Oral antibiotics with early hospital discharge compared with in-patient intravenous antibiotics for low-risk febrile neutropenia in patients with cancer: a prospective randomised controlled single centre study. Br J Cancer.

[21] Paganini HR, Sarkis CM, De Martino MG, Zubizarreta PA, Casimir L, Fernandez C (2000). Oral administration of cefixime to lower risk febrile neutropenic children with cancer. Cancer..

[22] Shenep JL, Flynn PM, Baker DK, Hetherington SV, Hudson MM, Hughes WT (2001). Oral cefixime is similar to continued intravenous antibiotics in the empirical treatment of febrile neutropenic children with cancer. Clin Infect Dis.

