# Supplementary material for: Early switch from intravenous to oral antibiotic therapy in patients with cancer who have low-risk neutropenic sepsis (the EASI-SWITCH trial): study protocol for a randomised controlled trial
Source: Trials. 2020 May 27;21:431. doi: 10.1186/s13063-020-04241-1 (PMC7251886; doi:10.1186/s13063-020-04241-1)
Supplement: Supplementary file 2 — Additional file 2. Patient information sheet. [file 13063_2020_4241_MOESM2_ESM.pdf]

<Site logo>

Early switch to oral antibiotic therapy in patients with low risk neutropenic sepsis

The **EASI-SWITCH** Trial

PATIENT INFORMATION SHEET

We would like to invite you to take part in our research study.

- Before you decide we would like you to understand why the research is being done and what it would involve for you.
- A study doctor or research nurse will go through this information sheet with you. Please ask questions about anything that might not be clear.
- Please take time to read the information carefully and discuss it with family and friends if you wish.
- Take your time to make your decision about whether or not to take part.

Thank you for reading this

**Page**	**Contents**
2	Information about why the study is being done and why you have been invited to take part
3-6	Detailed information about what would happen if you decided to take part
7	Study Calendar
8	Study flow chart
9	The pros and cons of taking part
10-11	Detailed information regarding confidentiality, study results and what to do if things go wrong
12	Important telephone numbers if you have questions about the study or are taking part and feel unwell at home

Why is this study being done?

Neutropenic refers to a low number of neutrophils, a special type of white blood cell important for fighting infection. Neutropenic sepsis is infection that may develop when there is a low white blood cell or neutrophil count. It is a common complication of chemotherapy treatment in cancer patients because in addition to killing cancer cells, chemotherapy also affects the bone marrow which makes new blood cells including neutrophils.

Patients can potentially become very unwell if an infection is left untreated when they have a low white blood cell count as they don't have the normal immune response to fight the infection. It is therefore important that patients are treated urgently with antibiotics to try and start fighting the infection immediately. Treatment normally involves intravenous antibiotics (through a drip) for several days.

We know, however, that most people are not at high risk of having a serious infection which makes them very unwell. Patients can be identified as 'low risk', by doctors calculating a special score, known as a MASCC score when they first arrive in hospital.

There is some evidence from small studies that 'low risk' patients can be safely switched from intravenous to oral (tablet) antibiotics within the first day of treatment.

Studies so far have not been rigorous enough to inform current treatment. We want to know for sure whether low risk neutropenic sepsis patients do not need as long a course of intravenous antibiotics – i.e. That an early switch from intravenous to oral antibiotics within the first day of treatment is as effective as intravenous antibiotics for a longer time.

Why have I been invited to take part?

You are being asked to take part in this study because you have a low white cell count and a suspected infection which requires antibiotic treatment. We believe you are at low risk of a serious medical complication from your infection and therefore suitable for either intravenous or oral antibiotics.

Do I have to take part?

No. It is up to you to decide whether or not to take part. If you decide to take part you will be given this information sheet to keep and asked to sign a consent form. You may still decide to withdraw at any time without giving a reason. If you decide not to take part, or if you withdraw, this will not affect your legal rights or healthcare at any time, now or in the future.

What are the alternatives for treatment?

If you decide not to enter this study you will receive the standard antibiotic treatment for neutropenic sepsis. It is your decision whether you would like to take part.

What will happen if I take part in this research study?

If your medical history and routine blood tests show that you can be in the study, and you choose to take part, then we will ask you to;

- Provide one additional blood sample
- Keep a diary at home
- Complete three short questionnaires and two short telephone interviews

You will **NOT** be able to participate if you;

- Are not in the low risk group.
- Have low blood pressure.
- Are suspected by your hospital doctor to have a severe infection.
- Have had previous difficulty taking or an allergy to the tablet antibiotics i.e. Penicillin antibiotics, Co-amoxiclav or Ciprofloxacin.
- Have been taking any medicines that would exclude you from the study.
- Are unable to take tablets.
- Are pregnant or breastfeeding

We will also check with your hospital doctor that they are happy for you to take part.

What happens once I consent?

If all your questions about the study have been answered and you are happy to take part, the study doctor will ask you to sign the study consent form.
The study doctor or research nurse will then do the following:

- Ask you about your medical history, cancer diagnosis, chemotherapy treatment and current medication.
- Take the research blood sample (12 mls/3 teaspoons) which will be stored for additional research tests.
- Ask you to complete a short quality of life questionnaire which relates to how you are feeling physically and emotionally and will take about 10 minutes to complete.
- Record your vital signs

You will have already been started on intravenous antibiotics when you were admitted to hospital.

To get a reliable result as to whether early oral antibiotics are just as effective as longer intravenous antibiotics we need to make a fair comparison and this is done by putting patients into two different antibiotic treatment groups using a technique called randomisation. The two antibiotic groups are:

1. **Standard intravenous antibiotic treatment.**
2. **Early switch oral antibiotics.**

A computer program selects which antibiotic group you would be in and you will be told the result. Neither you nor your doctor can choose your group.

For this study you have a 50% chance of being in the group receiving earlier oral antibiotics.

Just over 200 patients will join the study over the next three years from hospitals across the UK.

Whichever group you are in, you will still be looked after by the same team of doctors and nurses that are already treating you. You will continue to have the same routine care, including blood tests and physical examinations that you would have if you were not in this study. No further additional blood tests will be required for this study after the initial research blood sample is taken.

Antibiotic treatment

You will receive the antibiotic treatment for the group you have been allocated to.

If you are receiving **standard intravenous antibiotic treatment** you will continue to receive intravenous antibiotics for at least two days.
You will receive the standard intravenous antibiotics your hospital normally uses to treat neutropenic sepsis e.g. Tazocin or Meropenem.
The doctor looking after you will decide how long you need to stay on intravenous antibiotics and if/when you can switch to oral antibiotics.
If laboratory tests suggest a different antibiotic would be better for your infection or your temperature isn't quickly settling your doctor will change your antibiotics and explain to you their reasons for doing so.

If you are receiving **early switch oral antibiotics** you will switch over at 12-24 hours of having intravenous antibiotics to two oral antibiotics -
Ciprofloxacin twice a day and Co-amoxiclav three times a day to complete at least 5 days of antibiotic treatment in total.

All of the antibiotics used in this study are currently routinely used in the UK and no experimental drugs will be used. If your temperature doesn't settle within 2 days (48 hours) of switching to oral antibiotics (i.e. within three days of starting antibiotics) we will conclude that the early switch to oral antibiotics has not worked well enough at treating your infection. Your doctor may then change your antibiotics depending on what they think is the best treatment for you and will discuss this with you.

Your treating doctor will decide when you are ready to go home and give you clear advice as to what antibiotic treatment needs completed at home.

Diary

You will be given a patient progress diary to complete when you get home from hospital. This needs to be filled in until 14 days (2 weeks) after you first came into hospital.

We will ask you to record:

- Your temperature any time you feel unwell or feverish. Please use the thermometer you were given by your hospital when you started chemotherapy.
- All doses of oralantibiotics taken.
- Any possible side effects from the antibiotics.

- Any new medicines you start.
- Other significant events including if you are readmitted to hospital, develop a further infection or require further antibiotics.

If you have been discharged home and feel unwell or develop a temperature (38^{0}C or higher) you should immediately contact your hospital on the number you were provided with when you started chemotherapy. It is ESSENTIAL the hospital knows about any further temperatures or if you are generally feeling less well any time of the day or night. You may need to come back to hospital quickly to be seen by doctors.

If there are signs the infection is not being successfully treated your medical team will quickly organise any additional tests or treatments that you require. You may have to be readmitted for further antibiotic treatment.

Follow up interviews and questionnaires

We want to check on your progress at the end of the second week (Day 14 +/- 1 day) and fourth week (Day 28 +/- 1 day). If you have a hospital visit at these times, the research nurse will meet you for a short interview lasting approximately 15 minutes. If you do not have a visit scheduled then the research nurse will contact you by telephone.

On Day 14 they will check on your progress and review your diary with you. They will check in particular whether you

- Completed the planned course of antibiotics.
- Changed antibiotics or whether any further antibiotics were required.
- Had any possible side effects/complications from your antibiotic treatment.
- Had any further temperatures at home and what action was taken.
- Have started any new medications.
- Were readmitted to hospital.

The study research nurse will also review your medical notes to help answer these questions, particularly if you have required further medical assessments since you were discharged home. They may also contact your GP practice to help answer these questions also.

We will ask you to complete the same quality of life questionnaire you completed at the beginning of the study as well as one further short questionnaire to ask about your overall experience with the treatment you received. We will give you a copy of these questionnaires with this information sheet so if you think it would be helpful you can read these questions during the telephone call. We will need you to return the diary you kept and will ask you to post it in the stamped addressed envelope we gave you when you started the study.

At Day 28 the research nurse will again check on your progress and in particular whether you have been readmitted back into hospital with infection and the progress you have made restarting your chemotherapy.

The study calendar (see next page) shows what will happen to you during the study.

What happens when my involvement in this study stops?

You will continue to be looked after by your normal doctors. If you require further antibiotics for another infection you will not be asked to take part in the study again and the antibiotics you will be given will not be influenced by this study.

Study Calendar

Day/week	What you do
**After admission to hospital**	Meet your doctor and study team.<br>Receive Patient Information Sheet.<br>If agreeable, provide informed consent for:<br>(a) Research blood sample.<br>(b) Taking the antibiotic treatment for the study group you are allocated to.<br>Once you have consented you will-<br>• Get the required study tests and provide a blood sample.<br>• Have your medical history and medications checked.<br>• Complete a short quality of life questionnaire.<br>• Be told which antibiotic treatment group you have been allocated to.
**During your hospital stay**	Receive the antibiotic treatment you have been allocated.<br>If your doctor doesn't think your antibiotic treatment is working they will make changes.<br>Your doctor will discharge you home whenever they feel you are ready.
**Post discharge from hospital Weeks 1 - 4**	Take any remaining oral antibiotics.<br>Complete the progress diary until Day 14.<br>Check temperature if you feel unwell or feverish.<br>Telephone hospital if you fell unwell or have a temperature.
**Day 14**	Telephone interview with study research nurse who will check on your progress and go through two short questionnaires.<br>Complete and return your diary.
**Day 28**	Telephone interview with study research nurse.

Study Flow Chart

Another way to find out what will happen to you during the study is to read the study flow chart below. Start reading at the top and read down the list, following the lines and arrows.

Consent to take part

↓

Medical history and examination
1 research blood sample (3 teaspoons)
1 Short questionnaire

↓

Randomisation to 1 of 2 study groups

STANDARD ANTIBIOITCS

Continue with intravenous antibiotics for at least 48 hours
Decision to stop or switch to oral antibiotics made by your treating doctor

EARLY SWITCH ANTIBIOTICS

Start intravenous antibiotics and switch to two oral antibiotics 12-24 hours after starting intravenous antibiotics
Complete at least 5 day course of antibiotics

Discharge home from hospital when your treating doctor is happy

↓

Complete progress diary
Contact hospital if unwell

↓

DAY 14
Telephone interview - Complete 2 questionnaires
- Review and return diary by post

↓

DAY 28
Telephone interview

What are the possible benefits of taking part?

The overall aim of the study is to provide information about how effective an early switch to oral antibiotics is in low risk cancer patients who present to hospital with neutropenic sepsis. There is no guarantee you will directly receive added benefits from the treatment you receive in this study – we are trying to find out the best treatment for future patients who have infections while on chemotherapy.

If you are in the group receiving the standard antibiotics you will be receiving the same antibiotic treatment most patients receive when they present to hospital with neutropenic sepsis.

If you are receiving the oral antibiotics at an earlier stage we believe this is as effective and safe but this cannot be guaranteed. We also believe it may have several advantages for patients:

- Your quality of life may improve.
- There may be less complications or difficulties with intravenous lines.
- You may potentially get home from hospital sooner.

Analysis of the research blood samples will allow us to explore whether there are specific blood tests that can predict how severe an infection is or whether antibiotic treatment is going to work. You will not directly benefit from this but we hope that it will benefit future patients. These blood results will not be available to you or affect your care when you donate them.

What are the possible disadvantages and risks of taking part?

Potential risk of antibiotic treatment failure

There is a risk for patients in the early switch oral antibiotic group that their infection may not resolve as quickly compared with those who continue on intravenous antibiotics. This might require a change of antibiotics or switch back to intravenous antibiotics. If patients have been discharged home quickly there is a small chance they may need to come back to hospital and possibly be readmitted if doctors feel their infection is not settling or they are becoming more unwell.

Potential side effects of antibiotic treatment

The various antibiotics used to treat your infection all have different side effects, which the doctor looking after you will explain. In general oral antibiotics are more likely to cause nausea and vomiting than antibiotics given by injection. Both oral and intravenous antibiotics may cause diarrhoea or rash.

The initial insertion of an intravenous line, if you don't have a permanent line in place, involves some discomfort but this is usually mild. Intravenous lines have a small risk of becoming infected themselves and very occasionally can cause irritation or blood clots in the vein. If this happened the line would be removed. It is important to remember these risks are no different whether you take part in the study or not as it is currently standard practice to give antibiotics intravenously for neutropenic sepsis.

If you have any side effects or complications that may be related to an antibiotic or an intravenous line, you must tell the doctors or nurses looking after you.

Blood sampling

You will have one additional blood sample for this study taken in the normal way from a vein. You may feel mild discomfort and could experience slight bruising around the site where the sample is taken.

What will happen if I don't want to carry on with the study?

You can withdraw from the study at any time without giving a reason and your care will not be affected. You would continue to be looked after by your normal team of hospital doctors and nurses and receive the treatment they felt was best for your infection. If you withdraw from the study, we would use the data collected up to your withdrawal.

What if something goes wrong?

The NHS hospital remains responsible for your care during the study. If you have a concern about any aspect of the way you have been approached or treated during the course of this study, please speak to the study doctor or member of the research team for more information. If you remain unhappy and wish to complain formally, you can do this through the normal complaints service for your hospital, the contact details of this service are as follows: <INSERT CONTACT DETAILS FOR COMPLAINTS SERVICE AT LOCAL HOSPITAL>.

In the event of suffering some harm as a result of taking part in this study, due to someone's negligence, then you may have grounds for a legal action for compensation but you may have to pay your legal costs.

Will information from the study be kept confidential?

All information collected for the study will be kept strictly confidential. Your name will not be used in study report forms or results. With your permission, we will keep your GP informed about your participation in the study. Your medical notes and any data collected during the study may be inspected by responsible individuals from the local hospital, trial co-ordinating centre, sponsor or regulatory authorities.

What will happen to the results of the research study?

The study will take a number of years to complete. All the information will be published in medical journals and presented to other doctors and nurses at meetings and conferences. You will be able to request a summary of the main results if you wish. Names of patients will not appear in any reports or publications arising from the study.

What will happen to the research blood samples I give?

Research blood samples will be sent, with your consent, to the Centre for Cancer Research and Cell Biology (CCRCB) at Queen's University, Belfast. We are asking you to donate these samples for current use and/or long term storage and use in future research. The main aim of this part of the study is to see whether new blood tests might help predict how severe an infection is or how likely it is to respond to different antibiotic treatments. This research takes a long time and requires samples from many people before results will be known; results may not be available for many years.

Who is organising and funding the research?

The study is being sponsored by the Belfast Health and Social Care Trust and funded by the National Institute for Health Research Health Technology Assessment Programme. The study is organised by local researchers (contact details below) who do not receive payment for undertaking this research. There are no payments or reimbursement of expenses available to study participants.

Who has reviewed the study?

To protect your interests, all research in the NHS is looked at by an independent group of people called a research ethics committee. Ethical approval for this study has been obtained from a national ethics committee. Your local NHS trust has given approval for the study to take place at your hospital.

Will my GP be informed of my participation in the study?

With your permission we will inform your GP of your participation in the study and may be asked to provide information about you e.g. hospital admission and antibiotic prescription details.

Who to contact for further information

If you are taking part in the study, feel unwell at home or have a temperature it is important you immediately contact your hospital on the number you were provided with when you started chemotherapy.

For specific questions about the study you may contact your research doctor or nurse during work hours using the contact details below:

Study Doctor: ___________________________

Telephone number: ____________________________

Study Research nurse: ____________________________

Telephone number: ____________________________

Thank you for reading this information sheet and considering taking part in this study.

NHS
National Institute for
Health Research
