# Supplementary material for: Early switch from intravenous to oral antibiotic therapy in patients with cancer who have low-risk neutropenic sepsis (the EASI-SWITCH trial): study protocol for a randomised controlled trial
Source: Trials. 2020 May 27;21:431. doi: 10.1186/s13063-020-04241-1 (PMC7251886; doi:10.1186/s13063-020-04241-1)
Supplement: Supplementary file 3 — Additional file 3. Patient consent form. [file 13063_2020_4241_MOESM3_ESM.pdf]

<Site logo>

Early switch to oral antibiotic therapy in patients with low risk neutropenic sepsis

The **EASI-SWITCH** Trial

PATIENT CONSENT FORM

Patient's name: ____________________________

Principal Investigator name: ___________________________

Site name: ____________________________

Please initial each box

1. I confirm that I have read and understood the Patient Information Sheet for the above study dated xx/xx/xxxx (Version x.xx) and have been given a copy to keep. I have had the opportunity to ask questions and discuss the study. I understand why the research is being done and any foreseeable risks involved. ☐

2. I understand that my participation is voluntary and that I am free to withdraw at any time, without giving any reason, without my medical care or legal rights being affected. ☐

3. I understand that my medical notes and data collected during the study may be looked at by responsible individuals from the hospital, trial co-ordinating centre, sponsor or regulatory authorities, where it is relevant to my taking part in this research. I give permission for these individuals to have access to my records. I agree to information related to this research being retained at the Belfast Health & Social Care Trust. ☐

4. I give permission for information about me to be analysed in strict confidence by responsible people from the study team. ☐

5. I give permission for health checks and study blood tests to be carried out by nurses/doctors from the study team. ☐

6. I give permission for a blood sample to be taken, stored and analysed during this study and in the future for ethically approved research. Blood samples will be transferred, stored and analysed in The Queen's University of Belfast. Data generated will be used in strict confidence by responsible members of the study team. ☐

7. I agree to the data I provide being used in an anonymised format in publications and at conferences and understand that I will not be personally identified. ☐

8. I agree to my GP being informed of my participation in the study and for information about me to be provided to the research team. ☐

I agree to take part in the above study

______________________________	____________________	_____________
**Name of Patient**<br>**(Block capitals)**	**Signature**	**Date**
______________________________	____________________	____________
**Name of Person taking consent**<br>**(Block capitals)**	**Signature**	**Date**

1 copy for patient
1 copy for hospital notes
1 original for study site file
